# Stability and change in health behaviours as predictors for disability pension: a prospective cohort study of Swedish twins

**DOI:** 10.1186/1471-2458-11-678

**Published:** 2011-08-31

**Authors:** Annina Ropponen, Jurgita Narusyte, Kristina Alexanderson, Pia Svedberg

**Affiliations:** 1 Ergonomics, Institute of Biomedicine, Faculty of Health Sciences, University of Eastern Finland, Kuopio, Finland; 2 Division of Insurance Medicine, Department of Clinical Neuroscience, Karolinska Institutet, Stockholm, Sweden

## Abstract

**Background:**

Stability or changes of health behaviours have not been studied in association with incidence of disability pension (DP). The aims were to (1) investigate if stability or changes in health behaviours predict DP due to musculoskeletal diagnosis (MSD), (2) to evaluate if an association exists for DP in general, and (3) after taking familial confounding into account.

**Methods:**

The study sample was 16,713 like-sexed twin individuals born in Sweden between 1935-1958 (6195 complete twin pairs) who had participated in two surveys 25 years apart, were alive, and not pensioned at the time of the latest survey. Cox proportional hazards analysis was used to assess the associations (hazard ratios (HR) with 95% confidence intervals (CI)) between stability and change in health behaviours (physical activity, tobacco and alcohol use, body mass index (BMI)), and number of pain locations collected at two time points 25 years apart and the incidence of DP until 2008.

**Results:**

During the follow-up, 1843 (11%) individuals were granted DP with 747 of these due to MSD. A higher proportion of women were granted DP than men. Increase in BMI and stable use of tobacco products were predictors for DP due to MSD (HR 1.21-1.48) and DP in general (HR 1.10-1.41). The stability in the frequency of physical activity and increased frequency of physical activity were protective factors for DP due to MSD only when accounting for familial confounding. However, the number of pain locations (stability, increase, or decrease) was the strongest predictor for future DP due to MSD (HR 3.69, CI 2.99-4.56) and DP in general (HR 2.15, CI 1.92-2.42). In discordant pair analysis, the HRs for pain were lower, indicating potential familial confounding.

**Conclusions:**

Health behaviours in adulthood, including an increase in pain locations were associated with the incidence of DP. The association between physical activity and DP was especially related to adulthood choices or habits, i.e., the individual decision about frequency of exercising. Thus, it is important to e.g. increase public awareness of the potential beneficial effects of exercise throughout life to avoid permanent exclusion from the labour market for medical reasons.

## Background

Musculoskeletal disorders (MSD) are a major burden, not only to the individual because of reduced work capacity but also to the health care sector and thus the society as a whole since they incur high costs [1]. The common characteristics of all MSD are pain and reduced physical function [2,3]. Moreover, different MSD often coexist [4]. The social consequences of these disorders can be severe, and MSD are the largest diagnostic category accounting for award of disability pensions (DP). DP is the definitive outcome of work incapacity, leading to permanent exclusion of the individual from the labour market with major consequences for the individual, and not only in terms of reduced income [5]. However, there remains a need for understanding the predictors of DP due to MSD, since so far most of the studies have focused on DP in general [6-8] or on rather specific MSD diagnoses, such as back disorders [9].

Previous research has indicated that various health behaviours may influence the incidence of DP due to MSD. For example, several health behavioural factors (such as physical activity and diet) have been demonstrated to increase the risk for osteoarthritis, one of the main diagnoses within the group of MSD [10,11]. Moreover, overweight has been shown to be associated with increased risk of DP in general [7]. However, obesity or sport participation can also be considered as preventive or modifiable factors [10,11] since another study found that lack of physical fitness did not necessarily contribute to chronic conditions, such as MSD [12]. Although regular physical activity is one of the most common public health recommendations, the most beneficial dose of physical activity (such as the extent of regularity: exercise occasions daily, weekly, and should the regularity last for months, years or such) to prevent MSD, and hence to reduce the consequences of MSD such as DP, remains to be established [13].

An issue of concern is that any changes in health behaviours that occur at different times during an individual's life-time may have an impact on the MSD. Nevertheless, adults tend to be relatively conservative (stable) in their health behaviour very few changes tend to occur over the years. Both body mass index (BMI) and physical activity are known to be rather stable health behaviours [14,15]. Furthermore, experiences of musculoskeletal pain sites tend to be stable during the lifetime [16]. Moreover, the duration of specific health behaviours may influence the development of MSD. In other words, certain behaviours lasting only a short period or at irregular low levels such as smoking and drinking alcohol every now and then at parties, or smoking only for a short period, may not have similar effect as more prolonged exposure (such as regular smoking or alcohol consumption for many years) on MSD and their social consequences, such as DP.

A recent study revealed that genetic factors account for about 37% of the variation of DP due to MSD [17]. Environmental factors not shared by co-twins, such as adulthood experiences or choices, or habits of health behaviours (e.g., leisure-time activities and smoking), explained the remainder (63%) of the variation in DP due to MSD. The relatively large influence of environmental factors unique to each twin individual implies that risk factors for DP due to MSD can be modified. A twin study with a sufficient number of twin pairs discordant for DP due to MSD would be an effective tool to investigate whether health behaviour, stable or altered, could modify the risk for DP due to MSD. As far as we are aware, no such study has been conducted to date. Here we have examined the associations between stability or change in health behaviours and future DP due to MSD in a setting which can control for familial confounding.

This study aimed to investigate whether health behaviour, and changes in these behaviours, could predict DP due to MSD, taking familial confounding into account. Another aim was to evaluate if such an association also existed for DP in general.

We hypothesised that a stable pattern of health related behaviour may have an impact on the risk of DP due to MSD and DP in general. However, a change in behaviour may also affect later risk of DP. Therefore, both stability and change in a number of health-related behaviours was investigated, such as use of tobacco products (i.e., cigarette and pipe smoking and oral tobacco), alcohol consumption (irrespective of alcohol type and frequency), frequency of physical activity, and BMI, as well as number of pain locations.

## Methods

A prospective twin cohort study was conducted.

### Participants

Data from a prospective twin cohort study called STODS (Swedish Twin study Of Disability pension and Sickness absence) was used [18]. Data about twins born between 1925 and 1958 (N = 59 893) from the Swedish Twin Registry (STR) were available for this study. The first selection criterion for the study cohort was that a twin had participated in both the mailed questionnaire in 1973 and the telephone interview, Screening Across the Lifespan Twin study (SALT), conducted between January 1998 and March 2003 [19]. Second, only like-sexed twins with known zygosity were included. Third, individuals who at the time of the SALT interview were alive, living in Sweden, < 65 years old, and not retired (not on old-age or DP) were included. Hence, the final study sample consisted of 16,713 individuals born 1935-1958, including 6195 complete twin pairs of whom 2659 were monozygotic (MZ) and 3536 were dizygotic (DZ) twin pairs. Fifty-two per cent of the study sample was women. The questionnaire conducted in 1973 and the SALT-interview have been described in more detail elsewhere [19].

### Data sources and variables

The STODS data were obtained from the following sources. Data on date of birth, zygosity, sex, pain, and health behaviours from the two above mentioned two surveys were obtained from the STR [19]. Data on DP was collected from the MiDAS-database, the National Social Insurance Agency. Information on education, employment status, year of emigration, and old-age retirement was gathered from Statistics Sweden. Finally, data regarding date of death were obtained from the National Board of Health and Welfare. Data from the different national registers were linked together by using the unique ten digit Swedish identification number of each twin individual.

### Follow-up time and outcomes

**Figure 1:**
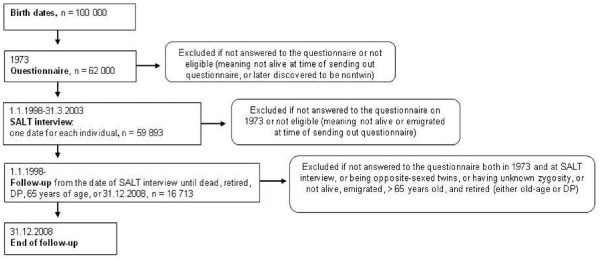
**Flow-chart of study time line**.

The following information about *DP *from the MiDAS-database was used: date granted DP and diagnosis according to ICD-10 [20] from the date of the SALT interview (between 1 January 1998 and 31 March 2003) until 31 December 2008 for all twins in the cohort (Figure 1). DP diagnoses were categorized as MSD (determined by ICD-10 rubrics M00-M99) and DP in general (that is, due to all diagnoses, including MSD). Individuals' data were the time until the age of 65, year of old-age retirement if that was before 65, emigration, date of death, or until end of follow-up at 31 December 2008.

### Exposures

Information on exposures was collected from the questionnaire in 1973 and from the SALT interview study, conducted approximately 25 years later (Figure 1). Exposures were categorized into groups of stability and change between the two time points as follow:

#### Leisure-time physical activity

Frequency of leisure time exercise (intensity and duration unknown) was enquired for with questions of exercise during a year: Do not exercise at all, Exercise very little, Exercise rather little, Exercise not that much, Exercise rather much, Exercise much, and Exercise very much. These were coded into four groups: 1) those who were frequent exercisers at both 1973 and SALT (stable frequent exercisers), 2) those who did little or no exercise (stable sedentary), and 3) those who increased, or 4) decreased the amount of exercise after 1973.

#### Body mass index (BMI)

BMI was calculated using self-reported weight and height information [(weight in kilograms/(height in meters) × (height in meters)]. Limits for underweight (< 18.5), normal weight (18.5-25), overweight (25-30), and obesity (> 30) were based on WHO standards [21]. Answers were coded into: 1) stable i.e. within same WHO category in 1973 and at SALT, 2) decreased i.e. decreased to a lower weight category after 1973, or 3) increased i.e. the individual had moved to a higher weight category after 1973.

#### Tobacco

Use of tobacco products, regardless of type (cigarettes, smokeless [oral] tobacco, pipe tobacco, and cigars) was enquired in both surveys and answers were categorized into three groups: 1) stable non-tobacco users (never), 2) stable tobacco users, and 3) those that changed their tobacco use behaviour after 1973 (either stopped, or started to use tobacco).

#### Alcohol consumption

Use of any type of alcohol (beer, wine, and spirits) was enquired in both interviews and categorized into two groups: 1) stable abstainers and 2) those that drank alcohol sometimes or regularly.

#### Number of pain locations

Questions about presence of back, shoulder, or neck pain were asked in both surveys and the questions were phrased as "Have you been suffering from severe pain in [then came the list] during the past years, resulting in difficulties to work?" Answers were coded as: 1) no pain at any of the surveys (never), 2) having the same pain at both surveys (stable), 3) decrease in number of pain locations after 1973, or 4) increase in number of pain locations after 1973.

### Background factors and covariates

*Zygosity *determination (MZ or DZ) for like-sexed twin pairs was obtained at the time of registry compilation on the basis of questions about childhood resemblance, and then updated during SALT for those twins not previously diagnosed or with uncertain diagnosis. When validated against serological and micro-satellite markers, this method is known to be 98% accurate [19].

Data on *employment status *(1970) and *years of education *(mean n years = 11) (at time of SALT) were obtained from Statistics Sweden.

Information on *marital status *was collected in the questionnaire 1973 and coded as "being married/co-habited" vs. "single, separated, or widowed".

#### Severity of diseases

Questions about the presence of common diseases and conditions were asked of all twins in SALT. Participants were asked to respond "yes" or "no" to the question "Do you have or have you had [here 53 different health problems and conditions were mentioned - listed in Table 1]?". In order to obtain a measure that reflects the medical severity of health problems instead of a summary variable of a number of self-reported conditions, Gold and co-authors' ratings of severity of health problems were applied to the SALT data [22]. An expert panel was asked to rate conditions in terms of their degree of being "life-threatening" according to the following three categories: "very life-threatening", "somewhat life-threatening", "and not at all life-threatening". For conditions available in SALT but not previously evaluated by Gold and colleagues, a four-member expert panel of physicians evaluated the conditions in terms of their degree of severity regarding the degree of being life threatening [23]. If there was no unanimity on the seriousness of a particular condition, the majority rating was used. Thereafter, a variable "number and severity of diseases" was created for each individual coded as "0", if no conditions at all were reported, "1", if conditions were "not at all life-threatening", "2", if any of the subject's conditions were "somewhat life-threatening" and, finally, "3", if any of the conditions present were "very life-threatening". If a person had several conditions, the code for chronic conditions was combined to represent these combinations (such as "Chronic conditions, both not at all life-threatening and somewhat life-threatening"). Altogether, eight categories were used, see Table 2.

**Table 1:** Included conditions categorized on the basis of the extent to which they can be considered as life-threatening (ratings by physicians)

Very Life-Threatening Condition	Somewhat Life-Threatening Condition	Not at all Life-Threatening Condition
Cardiac insufficiency	High blood pressure	Migraine
Angina pectoris	Vascular spasm in legs	Dizziness
Heart attack	Emphysema	Glaucoma
Thrombosis in leg	Chronic bronchitis	Cataract
Stroke	Tuberculosis	Other eye disorders
Multiple sclerosis	Allergies and Asthma	Knee disorders
Cancer	Epilepsy	Hip disorders
	Parkinson's disease	Psoriasis
	Mental conditions	Goiter/other gland disorders
	Polio	Stomach/intestinal disorders
	Rheumatoid arthritis	Gall disorders
	Osteoporosis	Gout
	Disabled (physically)	Urinary tract disorder
	Diabetes	Neck and shoulder disorders
	Ulcer	Eczema
	Liver disorders	Sciatica
	Kidney disease	Back disorders
	Prostate disorders	Scoliosis
	Uterus or ovary operation	
	SLE (Lupus)	
	Osteoporosis	
	Hay fever	
	Dyslipidemia	
	Cardiac arrhythmia	
	Cardiac murmur	

Modified from Svedberg et al. 2006. [22]

**Table 2:** Frequencies (percentages) of background factors and of health behaviours in the cohort specified for those later granted DP due to MSD, DP in general, and had no DP during follow up

	DP due to MSD				DP in general				No DP			
	Men		Women		Men		Women		Men		Women	
	MZ	DZ	MZ	DZ	MZ	DZ	MZ	DZ	MZ	DZ	MZ	DZ
Background factors	n (%)	n (%)	n (%)	n (%)	n (%)	n (%)	n (%)	n (%)	n (%)	n (%)	n (%)	n (%)
Employment at 1970												
≥ 35 hours	87 (84)	114 (79)	79 (41)	111 (36)	204 (77)	296 (75)	181 (38)	262 (37)	1646 (56)	2563 (57)	1062 (34)	1448 (33)
20-34 hours	1 (1)	-	17 (9)	37 (12)	3 (1)	-	40 (8)	61 (9)	23 (1)	26 (1)	172 (6)	267 (6)
1-19 hours	-	-	3 (2)	11 (4)	2 (1)	1 (0)	17 (4)	25 (4)	18 (1)	12 (0)	97 (3)	129 (3)
Student	13 (13)	22 (15)	33 (17)	53 (17)	38 (14)	72 (18)	115 (24)	147 (21)	1060 (36)	1664 (37)	1136 (37)	1506 (35)
Conscript in army	2 (2)	5 (3)	-	-	7 (3)	12 (3)	-	-	121 (4)	158 (4)	-	-
Other (not in paid work)	-	2 (1)	59 (31)	91 (30)	5 (2)	5 (1)	123 (26)	198 (28)	10 (0)	24 (1)	605 (20)	966 (22)
Missing data	-	2 (1)	2 (1)	1 (0)	4 (2)	8 (2)	3 (1)	10 (1)	35 (1)	49 (1)	22 (1)	30 (1)
Marital status												
Married or living with someone	90 (87)	120 (83)	145 (75)	232 (76)	213 (80)	306 (78)	339 (71)	510 (72)	2366 (81)	3696 (82)	2379 (77)	3366 (77)
Single, separated, widowed	14 (14)	25 (17)	48 (25)	73 (24)	52 (20)	88 (22)	140 (29)	193 (23)	551 (19)	803 (18)	723 (23)	981 (23)
Number and severity of diseases												
- None	1 (1)	2 (1)	1 (1)	1 (0)	2 (1)	12 (3)	1 (0)	1 (0)	162 (6)	247 (6)	105 (3)	160 (4)
- One or more not life-threatening	42 (40)	47 (32)	38 (20)	86 (28)	69 (26)	96 (24)	85 (18)	169 (24)	966 (33)	1637 (36)	999 (32)	1471 (34)
One or more somewhat life-threatening	2 (2)	2 (1)	1 (1)	5 (2)	4 (2)	13 (3)	6 (1)	11 (2)	127 (4)	218 (5)	109 (4)	137 (3)
One or more not and somewhat life-threatening	57 (55)	92 (63)	135 (70)	192 (63)	180 (68)	261 (66)	336 (70)	458 (65)	1562 (54)	2278 (51)	1657 (53)	2291 (53)
One or more very life-threatening	-	-	-	-	-	-	1 (0)	-	6 (0)	3 (0)	14 (1)	15 (0)
Two or more not and very life-threatening	-	-	2 (1)	7 (2)	1 (0)	2 (1)	7 (2)	12 (2)	26 (1)	33 (1)	63 (2)	101 (2)
Two or more somewhat and very life-threatening	-	-	-	1 (0)	-	-	1 (0)	3 (0)	3 (0)	8 (0)	9 (0)	8 (0)
Three or more not, somewhat and very life-threatening	2 (2)	2 (1)	16 (8)	13 (4)	9 (3)	11 (3)	42 (9)	49 (7)	65 (2)	73 (2)	145 (5)	164 (4)
**Health behaviour**												
Leisure-time physical activity												
Stable frequent exerciser	27 (26)	32 (22)	41 (21)	87 (29)	60 (23)	92 (23)	129 (27)	182 (26)	745 (26)	1162 (26)	1006 (32)	1334 (31)
Stable sedentary	19 (18)	34 (23)	51 (26)	70 (23)	51 (19)	93 (24)	112 (23)	181 (26)	598 (21)	857 (19)	646 (21)	955 (22)
Decreased the frequency of exercise	28 (27)	35 (24)	48 (25)	64 (21)	75 (28)	106 (27)	112 (23)	152 (22)	754 (26)	1243 (28)	643 (21)	927 (21)
Increased the frequency of exercise	8 (8)	13 (9)	25 (13)	46 (15)	22 (8)	27 (7)	63 (13)	96 (14)	244 (8)	395 (9)	476 (15)	683 (16)
BMI												
Stable	43 (41)	51 (35)	85 (44)	124 (41)	106 (40)	146 (37)	226 (47)	292 (42)	1139 (39)	1893 (42)	1462 (47)	2126 (49)
Decreased	-	1 (1)	1 (1)	2 (1)	4 (2)	6 (2)	5 (1)	12 (2)	21 (1)	39 (1)	19 (1)	49 (1)
Increased	46 (44)	72 (50)	78 (40)	137 (45)	113 (43)	185 (47)	188 (39)	300 (43)	1388 (48)	2016 (45)	1324 (43)	1692 (39)
Pain locations												
Never any pain	16 (15)	16 (11)	23 (12)	53 (17)	50 (20)	92 (23)	105 (22)	153 (22)	1136 (39)	1666 (37)	1291 (42)	1818 (42)
Stable number of pain locations	6 (6)	11 (8)	10 (5)	12 (4)	23 (9)	23 (6)	16 (3)	22 (3)	120 (4)	206 (5)	85 (3)	129 (3)
Decrease of pain locations	3 (3)	6 (4)	6 (3)	10 (3)	12 (5)	20 (5)	9 (2)	27 (4)	105 (4)	188 (4)	89 (3)	153 (4)
Increase of pain locations	66 (64)	88 (61)	127 (66)	196 (64)	142 (54)	196 (50)	291 (61)	409 (58)	1197 (41)	1884 (42)	1373 (44)	1828 (42)
Change of use of tobacco products												
Stable never user	9 (9)	9 (6)	26 (14)	50 (16)	24 (9)	35 (9)	76 (16)	109 (16)	367 (13)	441 (10)	677 (22)	880 (20)
Changed the use (increased or decreased)	19 (18)	21 (15)	40 (21)	56 (18)	41 (16)	69 (18)	91 (19)	131 (19)	795 (27)	1181 (26)	858 (28)	1057 (24)
Stable tobacco user	58 (56)	88 (61)	89 (46)	150 (49)	155 (59)	224 (57)	238 (50)	346 (49)	1390 (48)	2295 (51)	1245 (40)	1904 (44)
Alcohol consumption												
Abstainer	5 (5)	2 (1)	6 (3)	12 (4)	6 (2)	5 (1)	15 (3)	25 (4)	63 (2)	90 (2)	102 (3)	188 (4)
Sometimes or regularly	99 (95)	143 (99)	187 (97)	293 (96)	259 (98)	390 (99)	464 (97)	678 (96)	2854 (98)	4409 (98)	2999 (97)	4160 (96)

The study was approved by the Regional Ethical Review Board in Stockholm, Sweden (2007/524-31).

### Statistical analysis

Hazard ratios (HR) with 95% confidence intervals (CI) were computed using the Cox proportional hazards model with the follow-up time in days, and DP due to MSD and DP in general as the outcome variables. All Cox proportional hazard analyses were age adjusted. The analyses were clustered on twin pair identity to account for the fact that the twin pairs rather than independent individuals were sampled [24]. First, we calculated HR separately for MZ and DZ twins, and separately for men and women. Since the differences between zygosity or sex groups were negligible, we chose to run the analyses so as to include all individuals pooled together. However, to minimize the potential confounding of zygosity, all models were adjusted for zygosity. The analyses were stratified by sex. Each health behavior was analyzed separately, but we adjusted the models for: (1) education, to account for occupational and socio-economic status, (2) marital status, to account for family situation, and (3) the number and severity (level of life-threatening medical conditions) of diseases, to control for the potential effect of other chronic diseases on health behaviour (such as a cardiovascular disease preventing the individual from participating in physical activity or following the advice of a physician to stop use of tobacco products because of a disease) in the study.

Conditional Cox proportional hazard analyses were performed using twin pairs discordant for DP in general or DP due to MSD; i.e. one twin had been granted DP while the other twin had not been granted DP during the follow-up. The follow-up time to DP was evaluated in relation to the follow-up time of the co-twin. Familial confounding is evident in twin samples as twin pairs reared together share both their genetic make-up (100% for MZ and on average 50% for DZ twin pairs) as well as home and family environment. The potentially confounding familial factors can be controlled for by stratifying the sample by twin pair, which allows each twin pair their own baseline hazard. As a result, findings of associations between risk factors and DP in this analysis would not be explained by familial confounding. Statistical analyses were performed with Stata version 9.2 (Stata Corporation, College Station, TX, USA).

## Results

During a mean follow-up time of 6 years, a total of 1843 DP were granted (11% of all individuals) and 747 of those were due to MSD (41% of all DP). More women than men were granted DP in general (n = 1183 vs. 660, in percentages 64% vs. 36%) and due to MSD (n = 498 vs. 249, 27% vs. 14% of all diagnoses). The characteristics of the study sample are presented in Tables 2 and 3. Almost all individuals in the cohort were working at the time of the first interview in 1973, when they were between the ages of 16-48. Very few (0-6%) reported any chronic medical conditions at time of follow up, most (51-70%) had conditions that were considered only as somewhat life-threatening (see Tables 1 and 2).

**Table 3:** Means (standard deviations) of background factors for those granted DP due to MSD, DP, and those not granted DP in the twin cohort

	DP due to MSD				DP in general				No DP			
	Men		Women		Men		Women		Men		Women	
	MZ	DZ	MZ	DZ	MZ	DZ	MZ	DZ	MZ	DZ	MZ	DZ
Background factors	Mean (SD)	Mean (SD)	Mean (SD)	Mean (SD)	Mean (SD)	Mean (SD)	Mean (SD)	Mean (SD)	Mean (SD)	Mean (SD)	Mean (SD)	Mean (SD)
Follow-up time (years)	4 (2.0)	4 (2.2)	4 (2.3)	3 (2.1)	4 (2.1)	4 (2.2)	4 (2.2)	3 (2.2)	6 (1.7)	6 (1.7)	6 (1.7)	6 (1.8)
Age at SALT-interview	55 (3.8)	54 (4.4)	54 (4.6)	54 (4.8)	55 (3.9)	54 (4.6)	54 (4.5)	54 (4.7)	53 (5.6)	52 (5.7)	52 (5.8)	53 (5.8)
Education (years)	10 (1.8)	10 (1.9)	11 (2.2)	10 (2.1)	11 (2.2)	10 (2.3)	11 (2.5)	11 (2.3)	12 (2.6)	11 (2.5)	12 (2.5)	11 (2.5)

With respect to health behaviours, there was no clear pattern in the risk for DP. Increase in BMI (HR 1.21, 95% CI 1.03, 1.41) and stable use of tobacco products (HR 1.48, 95% CI 1.17, 1.85) over the 25 years were both independent predictors for DP due to MSD and for DP in general (HR 1.10, 95% CI 0.99, 1.21, HR 1.41, 95% CI 1.22, 1.62, respectively). A decrease in BMI was also associated with an increased risk for DP (HR 1.58). These associations of BMI and of use of tobacco products with DP did not remain significant when familial confounding was taken into account. Low physical activity, i.e., sedentary behaviour, or increased frequency of exercise exerted a protective effect on DP due to MSD only when taking into account familial confounding. Stability, increase, and decrease of number of pain locations between the two time points were the strongest predictors for future DP due to MSD (HR 2.32-3.69) and DP in general (HR 1.72-2.15) (Table 4). The results of conditional Cox proportional hazard accounting for familial confounding confirmed these findings.

**Table 4:** Cox proportional hazard ratios (HR) with 95% confidence intervals (CI) of each health behaviour for those granted DP due to MSD and DP in general

	DP due to MSD				DP in general			
	Whole sample		Discordant pairs		Whole sample		Discordant pairs	
Health behaviour	HR*	95%CI	**HR**^#^	95%CI	HR*	95%CI	**HR**^#^	95%CI
Leisure-time physical activity								
Stable frequent exercisers (reference group)	1.00		1.00		1.00		1.00	
Stable sedentary	1.09	0.88, 1.34	**0.63**	**0.44, 0.91**	1.13	0.99, 1.29	0.84	0.68, 1.04
Decreased the frequency of exercise	1.16	0.94, 1.43	0.99	0.69, 1.42	**1.17**	**1.03, 1.34**	1.13	0.92, 1.38
Increased the frequency of exercise	0.98	0.76, 1.25	**0.64**	**0.43, 0.94**	0.94	0.80, 1.10	0.84	0.66, 1.08
BMI								
Stable (reference group)	1.00		1.00		1.00		1.00	
Decreased	0.54	0.20, 1.45	1.34	0.31, 5.91	**1.58**	**1.07, 2.32**	1.30	0.63, 2.67
Increased	**1.21**	**1.03, 1.41**	0.96	0.73, 1.27	1.10	0.99, 1.21	1.12	0.95, 1.32
Pain locations								
Never any pain (reference group)	1.00		1.00		1.00		1.00	
Stable number of pain locations	**3.54**	**2.45, 5.13**	**2.65**	**1.29, 5.44**	**2.04**	**1.60, 2.59**	**1.46**	**1.01, 2.10**
Decrease of pain locations	**2.32**	**1.50, 3.57**	**2.98**	**2.09, 4.27**	**1.72**	**1.33, 2.21**	1.07	0.71, 1.60
Increase of pain locations	**3.69**	**2.99, 4.56**	1.51	0.74, 3.08	**2.15**	**1.92, 2.42**	**1.61**	**1.36, 1.90**
Change in use of tobacco products								
Stable never user (reference group)	1.00		1.00		1.00		1.00	
Changed the use (either increased or decreased)	1.16	0.89, 1.52	1.20	0.77, 1.86	1.04	0.88, 1.23	0.89	0.69, 1.14
Stable tobacco user	**1.48**	**1.17, 1.85**	1.23	0.75, 2.02	**1.41**	**1.22, 1.62**	1.19	0.89, 1.59
Alcohol consumption								
Abstainer (reference group)	1.00		1.00		1.00		1.00	
Sometimes or regularly	1.24	0.83, 1.86	0.49	0.20, 1.20	**1.34**	**1.02, 1.78**	0.80	0.50, 1.27

* All HR's are adjusted for zygosity, birth year, years of education, marital status, and number and severity of diseases. Sex was stratified allowing men and women their own baseline hazard

^# ^Conditional Cox proportional hazard

## Discussion

In this prospective cohort study of the incidence of DP among adult Swedish twins, 11% had been granted DP during the six year (on average) follow-up. Nearly half of the DPs were due to MSD and it was found that some health behaviour aspects and changes were associated with DP due to MSD as well as with DP in general. These results remained unchanged when adjusting for marital status, education, severity of diseases, and age - all well known factors affecting the incidence of DP. Familial factors do not seem to influence these associations thus suggesting that factors occurring during the adulthood play the predominant role, except for the association between physical activity and DP due to MSD.

One unexpected finding from this study, in contrast to prior evidence linking unhealthy behaviours to disease onset and disabilities [7,25], was that neither stability nor change in any other health behaviour (in our study alcohol consumption or change of use of tobacco products) displayed any clear-cut associations with future DP. Though, this is in line with a recent Finnish study of twins, where smoking measured at one time-point, but in 30 years of follow-up predicted higher risk for DP due to low back disorders, whereas alcohol consumption did not [26]. However, our lack of association between alcohol consumption (as tested "stable abstainers" as referent, and "those who drank alcohol sometimes or regularly" as being in risk) and DP is maybe partially related to the fact that we did not have such detailed information about alcohol consumption. Thus, we could not analyze this using a more detailed categorization. Since we were able to detect changes over time in health behaviour, we expected to see some impact of a change in BMI, use of tobacco products, or alcohol consumption. This was of particular interest since it is known that adults tend to be relatively stable in their health behaviour [14,15], in fact many public health recommendations are targeted to change these behaviours. Instead, our results point in the direction that health behaviours may be mediated by familial confounding, that is, by factors that are shared in childhood such as family environment and genes. As shown in Table 3, some increased risk of DP was related to an increase in BMI over time and to the stable use of tobacco products, but the associations were no longer statistically significant after accounting for familial confounding. Nevertheless, frequency of physical activity, either a stable sedentary behaviour or a change i.e. increased frequency of exercise showed a reduced risk for DP due to MSD when familial confounding was accounted for. There was no association when familial confounding was not taken into account. The association between physical activity and DP in general was also protective, though non-significant, after controlling for familial confounding. The effect of familial confounding indicates that some adulthood choices or habits can play an important role in physical activity for example; these may affect choices about the type and frequency of exercise, which in turn affects DP. If the adulthood choices or habits are really of importance for the association between physical activity and DP, as the result suggests, there might be possibilities to target public health campaigns to increase the public awareness of benefits of physical activity and/or to target interventions such that physical activities are promoted.

The number of pain locations between the two time points, separated by approximately 25 years, showed the strongest association with DP in this cohort of individuals in gainful employment at the time of the first data collection in 1973. At that time, almost all individuals in the cohort were working (Table 2). Both stability and an increase in the number of pain locations predicted DP in general as well as DP due to MSD occurring over an average 6 years of follow-up. This result also persisted after familial confounding was taken into account, suggesting that pain location is a characteristic associated to DP due to MSD, and is less likely to be affected by shared family or genetic factors. Previous studies of pain have shown pain to have a biological basis i.e. there is a genetic component (e.g., [27]). Although pain as a risk factor for DP was an expected finding, the predictive value of pain for future DP many years later is highly relevant for both the individual and public health as a whole. From a public health perspective, pain management [28] as a tool for health care providers should be considered.

This study used data from a population-based Swedish twin cohort and detailed DP information including the dates and diagnosis of DP. Further, strengths of the study include the large cohort, and collection of exposure data at two time points separated by a relatively long time period (25 years), enabling measurement of stability and change in health behaviour. In addition, there were no drop-outs regarding follow-up information. Moreover, a prospective study design was used with a relatively long follow-up (a maximum of 10 years) and the use of the twin setting made it possible to control for familial confounding. The access to twin data gives a unique opportunity to control for familial confounding by using co-twin control design. This design allows analyzing twins discordant for both outcome (in this case DP due to MSD) and exposure (such as health behaviour aspects and changes). The co-twin control design is a powerful tool since cases and controls are matched optimally being twins (monozygotic twins share on average 100% of their genes at the sequence level, and dizygotic twins 50% of their segregating genes). Moreover, the twin pairs are age-matched by definition and in the present study the pairs were also matched by sex. The analysis of outcome and exposure discordant twins investigates whether twins who are exposed to a specific risk factor more often are granted a DP than their non-exposed co-twins. One limitation was that several additional changes regarding exposures might have occurred between the first and second data collection, and these might have impacted on the observed associations. This cannot be ruled out in the study, but the use of two time-points before the beginning of follow-up of DP was done in order to gain some insight into this phenomenon. Although no detailed information about the duration of exposure to the health behaviours was available, we assume that the two time points used may reflect duration, at least at a crude level. To minimize this problem, only those twins who had participated in both surveys were included. The non-responders, that is, twins participating in 1973 but not in SALT, have previously been evaluated, and it was found that non-responders were similar to the responders at least in terms of how they had responded to the questionnaire in 1973 [19]. Another limitation is that our measure of BMI was based on self-reported weight and height. However, we may assume that our BMI should be a relatively reliable measure. A recent study also based on the Swedish twin registry found that self-reported and assessed height, weight, and BMI have significant and substantial between measurement agreements [29].

It is worth noting that the factors under study may be mutually related [30,31]. For example, pain may prevent an individual from exercising for shorter or longer time periods, or smoking cessation may be facilitated by motivation to improve one's fitness level. Similarly, one health-related behaviour (e.g., smoking) may have a mediating effect on the association between a second health-related behaviour (e.g., exercising) and DP. In this study, we have primarily focused on studying the stability or change of health behaviours and their effect on the development of DP. Therefore, we have intentionally excluded the analysis of between-health behaviours associations.

Since health behaviours may be influenced by the grade and the number of chronic conditions, we have also adjusted for severity of diseases in our analyses. One would anticipate that the severity of diseases (or number of chronic conditions) would also include information on pain to various degrees and in different regions. Pain seems to be a characteristic present in almost all MSD. Therefore, though a consideration of pain as an indicator of existing disease maybe a relevant issue for short-term follow-ups, with our data with its long follow-up time, it seemed more appropriate to analyze pain as an independent risk factor. Moreover, we may assume that by adjusting for severity of diseases, we have also captured some of the pain-related aspects into our analyses. The descriptive analysis of the sample showed that less than 9% of subjects with DP had very life-threatening conditions, whereas most individuals (55-70% of those with DP) suffered from conditions that were not at all life-threatening or conditions that were considered only somewhat life-threatening.

## Conclusions

Health behaviour in adulthood, such as long-term use of tobacco products, BMI increase, physical activity, and both stability and change in the number of pain locations has an effect on the incidence of DP. In particular, the effect of physical activity is related to adulthood choices or habits, not being affected by the childhood environment. Therefore, to keep a stable weight and to continue to be physically active throughout the lifespan, when possible, is of importance.

## Competing interests

The authors declare that they have no competing interests.

## Authors' contributions

AR and PS designed the study, carried out the statistical analysis and drafted the manuscript. AR led the critical review and revision of the manuscript. PS, JN and KA all contributed substantially to the interpretation of results, drafting and review of the manuscript. All authors have read and approved the final manuscript.

## Pre-publication history

The pre-publication history for this paper can be accessed here:

http://www.biomedcentral.com/1471-2458/11/678/prepub
